# Peer review in Clinical Pharmacology using the 8-D Assessment

**DOI:** 10.5414/CP202971

**Published:** 2017-02-14

**Authors:** Barry G. Woodcock

**Affiliations:** Dustri Medical Science Publications, Munich-Oberhaching, Germany

**Keywords:** peer review, clinical pharmacology, 8-D Assessment

## Abstract

The requirement for editors of clinical pharmacology journals to maintain an overview of the peer review process for manuscripts submitted can be facilitated by use of the **8-D Assessment.** The **8-D Assessment** comprises peer review criteria to determine if the:1. **D**esign of the study, 2. **D**iagnoses employed, 3. **D**rug molecules involved, 4. **D**osages applied, 5. **D**ata collected, 6. **D**iscussion of the findings, 7. **D**eductions made, and 8. **D**ocumentation are in accord with the objectives of the study and meet the requirements of evidence-based medicine. This tool, although easy to apply, requires a high level of clinical pharmacology expertise, especially in the fields of drug disposition, pharmacokinetics, and drug action.

Since medical research is not driven by altruism or an innate human need to alleviate suffering, the *peer review* of manuscripts reporting research findings must be transparent regarding a) the identity and background of the peer reviewer and b) how the peer review was done. 

The important aspects concerning the identity and background of the peer reviewer have been addressed recently in an article with the rather ominous title *“Organised crime against the academic peer review system”* [1]. The main message in this article was that some editors have lost the overview in managing manuscripts submitted to their journal. In some instances to the extent that authors have been able to “peer review” their own manuscripts as in the case of Lv, Deng, and Long in a paper published and subsequently withdrawn in the Br J Clin Pharmacol [2]. 

Information on the assessment procedures used in peer reviews is almost always unknown. Choice of assessment criteria is usually left to the discretion of the reviewer. Peer review criteria in the clinical field have been discussed from the ethical standpoint, but little attention has been given to defining exactly what these criteria should be [3]. 

In the case of clinical pharmacology, peer review criteria should be suitable for manuscripts covering a wide variety of topics, research protocols, and manuscript formats. As well as being clearly defined, comprehensive, and dealing with the aspects important in clinical pharmacology, they must simple to apply. The publication of clinical findings is a driving force in pharmacotherapy, and therefore the peer review process is a determinant for safety in drug development. The peer review process must therefore meet the requirements of Evidence-Based Medicine (EBM) [4]. 

Peer review criteria for clinical pharmacology were recently put forward by Woodcock and Luger [5]. This tool, now slightly modified, is described here. Although easy to apply, it requires a high level of clinical pharmacology expertise, especially in the fields of drug disposition, pharmacokinetics, and drug action. 

The **8-D Assessment** is used to determine if the:

1. **D**esign of the study,
2. **D**iagnoses employed,
3. **D**rug molecules involved,
4. **D**osages applied,
5. **D**ata collected,
6. **D**iscussion,
7. **D**eductions made, and
8. **D**ocumentation support

are in accord with the objectives of the study and meet the requirements of EBM where: 

1. *Right design* means that the study design and protocol are appropriate for answering the question(s) being asked. 

2. *Right diagnosis* is relevant for investigations both in patients and healthy subjects where subject and patient description and patient selection need to be detailed, accurate and appropriate for the aims of the study. 

3. *Right drug molecule* begs the questions “Is the active agent a known molecular species?” and “Can the drug entity have a mode of action compatible with the observed pharmacological effects? Has a pharmacological effect observed in vitro a counterpart in vivo? Do confounding factors exist such as the presence of drug enantiomers, stereoisomers, or drug combinations? Herbal drugs and extracts do not generally fit in with the concepts of EBM. High first pass effects make it likely that more than one active species is present in the tissues. 

4. *Right dosage* concerns not only the size of the dose or doses (i.e. Is the dose or concentration clinically relevant and safe according to the clinical, animal, and in-vitro data available?), but also the method of administration, bioavailability, and duration of treatment. These questions also apply to in-vitro studies with tissues and cells. 

5. *Right data* are those data required to meet the objectives of the study, which can establish or disprove efficacy, which have been obtained using state-of-the-art methods, and which have been evaluated using recognized data analysis procedures. In the case of reviews of the literature, the retrieval methods used and quality of the studies reviewed would need to be scrutinized. 

6. *Right discussion* means that all limitations of the study are stated, new findings are highlighted, differences compared to other investigations are discussed satisfactorily, and that due recognition is given to the work of earlier investigators in the field. 

7. *Right deductions* i.e. conclusions are based on a correct and objective interpretation of the research findings and that recommendations are made with due caution regarding patient safety and efficacy requirements in clinical pharmacotherapy. 

8. *Right documentation* addresses primarily the quality of the evidence in the supportive literature and asks the questions “Is the documentation up-to-date? Is it obtained from peer-reviewed sources and is it comprehensive?” The citation of websites is very useful for providing information, but must be viewed with caution when used to provide evidence. Information on websites is not peer-reviewed and can be subject to change. 

If the finding of any one of these assessments is questionable, the compliance of the research with EBM principles will be weakened. The reviewer has then the duty of making a list of comments and recommendations to the authors and editor accordingly. 

## Conclusions 

The **8-D Assessment** comprises peer review criteria to determine if the:1. **D**esign of the study, 2. **D**iagnoses employed, 3. **D**rug molecules involved, 4. **D**osages applied, 5. **D**ata collected, 6. **D**iscussion of the findings, 7. **D**eductions made, and 8. **D**ocumentation in a clinical pharmacology manuscript are in accord with the objectives of the study and meet the requirements of evidence-based medicine. This tool, although easy to apply, requires a high level of clinical pharmacology expertise, especially in the fields of drug disposition, pharmacokinetics, and drug action. 

## Conflicts of interest 

BGW is Editor-in-Chief of the International Journal of Clinical Pharmacology and Therapeutics published by Dustri-Verlag Dr. Karl Feistle GmbH & Co. KG, Munich-Deisenhofen, Germany and Inc., Rockledge, FL, USA. 
